# An Overview of Outcomes Associated With Early Versus Late Tracheostomy From a National Standpoint

**DOI:** 10.7759/cureus.16325

**Published:** 2021-07-12

**Authors:** Guiseppe Filice, Palak Patel, Priyaranjan Kata, Anish Kanukuntla, Vraj Patel, Neil Gallagher, Pramil Cheriyath

**Affiliations:** 1 Internal Medicine, Hackensack Meridian Health Ocean Medical Center, Brick, USA

**Keywords:** early tracheostomy, late tracheostomy, in-hospital mortality, outcome of early versus late tracheostomy, length of stay in the hospital

## Abstract

Tracheostomy is a procedure that is commonly used in critically ill patients who require prolonged mechanical ventilation due to acute respiratory failure or airway problems. The best tracheostomy timing (early vs. late) and techniques (percutaneous dilatational, other new percutaneous procedures, open surgery) have been hotly debated. This research aimed to evaluate the outcome of early versus late tracheostomy in terms of in-hospital mortality, patient length of stay in the hospital, and cost after a detailed analysis and review using National Inpatient Survey (NIS) data. This study indicates that early tracheostomy greatly reduces in-hospital mortality, the need to transfer to skilled nursing facilities as well as direct variables, length of stay, and potentially overall hospital cost in the ICU.

## Introduction

Tracheostomy is one of the oldest procedures performed in history, and it was first described as early as 100 B.C. by a Greek physician Asclepiades. A tracheostomy is normally carried out while clinicians expect an affected person will require prolonged assisted ventilation. The use of this procedure has increased, specifically following the creation of a realistic bedside percutaneous tracheostomy method in 1985 such that as much as one-third of patients requiring extended mechanical airflow now obtain a tracheostomy [1,2]. Improved patient comfort, less sedative medication usage, quicker weaning from artificial breathing, a lower incidence of nosocomial pneumonia, shorter hospitalization more effective tracheal suctioning, less anatomic dead space which is especially important in chronic obstructive pulmonary disease (COPD), and of course reduced incidence of vocal cord damage are all considered benefits of tracheostomy versus prolonged trans laryngeal endotracheal intubation [3]. Many physicians perceive that early tracheostomy might maximize these benefits during the course of illness. However, there is a concern over whether early tracheotomy is better than late tracheotomy or extended intubation [4]. The timing of a tracheotomy, on the other hand, was often determined by the physician's personal preferences, the patient's health status, and the patient's relative's opinion. During the past few years, studies have shown that early tracheostomy in intubated patients improves the overall outcome [5]. This research aimed to evaluate the outcome of early versus late tracheostomy in terms of in-hospital mortality, length of patient's stay in hospital, and cost.

## Materials and methods

This is a retrospective research aimed to evaluate the outcome of early versus late tracheostomy using National Inpatient Survey (NIS) data of the Healthcare Cost and Utilization Project

Data source

The National Inpatient Survey (NIS) is the nation's largest publicly accessible all-payer inpatient healthcare database, to produce regional and national assessments of inpatient use, access, charges, cost, and outcomes in the United States. The NIS is drawn from the State Inpatient Databases (SID), which contain all inpatient data currently submitted to the Healthcare Cost and Utilization Project (HCUP). The NIS is made up of data from all HCUP-participating states, accounting for more than 97% of the population of the United States. The NIS approximates a 20% stratified survey of all discharges from U.S. general hospitals, except outpatient and long-term intensive care hospitals, beginning with the 2012 data year. This dataset contains codes for both primary and secondary diagnoses, as well as procedures conducted during the hospitalization.

Study population

Using the NIS dataset, the patient who underwent tracheotomy during the hospitalization were included for our analysis. They were categorized into two types of tracheostomy, early tracheostomy (≤ 7 days) and late tracheostomy (between nine and 16 days).

Variables and outcome of interest

Database characteristics included gender, race, geographic region, Elixhauser comorbidities (which includes congestive heart failure, valvular disease, pulmonary circulation disease, peripheral vascular disease, paralysis and other neurological disorders, chronic pulmonary disease, hypertension, diabetes with and without chronic complications, renal failure, liver disease, coagulopathy, obesity, fluid and electrolyte disorders, alcohol abuse and deficiency anemias), hospital ownership/control setting. Disposition included discharge to home, transfer to other facilities, home health care, and discharge against medical advice. In-hospital mortality evidence is compared with early and late tracheostomy patients. The duration of stay (days) and gross hospitalization expense were factors in resource utilization.

Statistical analysis

The statistical analysis was performed using SAS 9.4 (Cary, NC: SAS Institute). NIS database from the fourth quarter of 2015, 2016, and 2017 was used for analysis. The continuous variables were expressed as a mean± SD and were compared using the Student t-test. Categorical variables were reported as a frequency (%) and compared using chi-square tests. All tests were considered significant when a p-value was less than 0.05. Identified two groups with early tracheostomy if tracheostomy was performed within seven days of admission and late tracheostomy if tracheostomy was performed between day nine to day 16 of the hospitalization using International Classification of Diseases 10th Revision (ICD 10) diagnostic codes and procedure codes. We Included all patients with tracheostomy performed during the same-day admission. The use of the logistic regression model included age, gender, race, Elixhauser comorbidities, hospital location, and hospital region. For the calculation of the cost of hospitalization, the cost to charge ratio files was utilized and merged with the original files. These files are provided by the sponsor National Inpatient Survey-Healthcare Cost and Utilization Project (NIS-HCUP). The final cost was calculated by multiplying the total cost with the cost to charge ratio. These results were included in the tables mentioned in this study.

## Results

There were a total of 127,475 tracheostomy procedures performed as per data collections. Early tracheostomy is ≤7 days, late tracheostomy is between nine and 16 days. Early tracheostomies were performed on a total of 55,875 (43.8%) patients, with 23,720 (41.4%) males, and 33,600 (58.6%) females. In comparison, 49,960 (39.2%) late tracheostomies were performed on 22,815 (44.2%) males and 28,820 (55.8%) females. We stratified the data of different factors like length of stay, hospitalization cost, Elixhauser comorbidities, geographic distributions, and hospital ownership that could be associated with disparity in tracheostomy timing in Tables 1, 2, 3, 4, respectively.

**Table 1:** Outcomes of early versus late tracheostomy on length of stay and hospital cost

	Early Tracheostomy	Late Tracheostomy
Length of stay (days)	27.7	32.3
Total hospitalization cost ($)	101,141	116,047

**Table 2:** Elixhauser comorbidities W/o: with or without

	Early Tracheostomy	Late Tracheostomy
Congestive heart failure	14,140 (24.7%)	15,075 (29.2%)
Valvular disease	1495 (2.6%)	1180 (2.3%)
Pulmonary circulation disease	2340 (4.1%)	2585 (5.0%)
Peripheral vascular disease	2865 (5.0%)	2455 (4.7%)
Paralysis	11,010 (19.2%)	9630 (18.6%)
Other neurological disorders	10,440 (18.2%)	9345 (18.1%)
Chronic pulmonary disease	12,710 (22.1%)	10,845 (21.0 %)
Hypertension	23,105 (40.3%)	19,705 (38.1%)
Diabetes w/o chronic complications	4245 (7.4%)	3530 (6.8%)
Diabetes w/ chronic complications	6070 (10.6%)	5885 (11.4%)
Renal failure	7575 (13.2%)	8095 (15.7%)
Liver disease	1695 (3.0%)	1880 (3.6%)
Coagulopathy	7730 (13.5%)	8165 (15.8%)
Obesity	8300 (14.5%)	7610 (14.8%)
Fluid and electrolyte disorders	34,265 (59.8%)	33075 (64.0%)
Deficiency anemias	6905 (12.0%)	6090 (11.8%)
Alcohol abuse	3630 (6.3%)	3360 (6.5%)

**Table 3:** Geographic distributions

Region	Early Tracheostomy	Late Tracheostomy
Northeast	8300 ( 14.5%)	9350 (18.1%)
Midwest	13,510 ( 23.6%)	10,855 ( 21.02%)
South	24,410 ( 42.6%)	21,035 (40.7%)
West	11,110 (19.4%)	10,400 ( 20.1%)

**Table 4:** Type of hospital ownership

Hospital Ownership/Control	Early Tracheostomy	Late Tracheostomy
Rural	1700 ( 3.0%)	1560 ( 3/0%)
Urban nonteaching	9254 (16.1%)	10,085 (19.5%)
Urban teaching	46,375 (80.9%)	39,995 (77.4%)

Outcomes

In terms of in-patient mortality, hospital discharge, and transfer to other skilled facilities, we compared the outcomes of early versus late tracheostomy. We compared the proportions for early and late tracheostomy using a Z-score, two-tailed hypothesis, and the difference is statistically significant with a p-value of less than 0.05. (Table 5). The differences in tracheostomy timing between ethnic groups are statistically significant (p < 0.05) for Caucasians, African Americans, and Hispanics, but not for Native Americans (p = 0.17384, p > 0.05) (Table 6).

**Table 5:** Outcomes of early versus late tracheostomy, disposition

	Early Tracheostomy	Late Tracheostomy	p-Value
In-hospital mortality	6205 (10.8%)	6935 (13.4%)	<0.0001 (significant)
Discharge to home	3900 (6.8%)	2265 (4.4%)	<0.0001 (significant)
Transfer to other: includes skilled nursing facility (SNF), intermediate care facility (ICF), and another type of facility	2615 (4.6%)	2555 (5.0%)	<0.00108 (significant)
Home healthcare	40,530 (70.7%)	37,685(73.5%)	<0.0001 (significant)
Against medical advice (AMA)	20(0.9%)	5(0.7%)	<0.00634 (significant)

**Table 6:** Disparity in tracheostomy timing in different ethnicities

	Early Tracheostomy	Late Tracheostomy	p-Value
Caucasians	34,365 (61.5%)	28,870 (57.8%)	<0.0001 (significant)
African American	11,260 (20.1%)	11,545 (23.1%)	<0.0001 (significant)
Hispanic	5534 (9.9%)	5444 (10.9%)	<0.0001 (significant)
Native American	4715 (8.4%)	4100 (8.2%)	=0.17384 (not significant)

## Discussion

Tracheostomy is a surgical procedure that establishes an airway in the trachea. It is most often used in patients who have had trouble weaning off a ventilator, intending to ease weaning by reducing work of breathing in patients with reduced reserve, to protect the vocal cords from damage induced by an endotracheal tube passing between them and exerting pressure on vocal cords, reducing the need for sedation, and its associated negative effects on blood pressure and gut function, and allowing for earlier patient mobilization, absorbing gastrointestinal feeds, and physical and occupational therapy [6]. In between 10% and 15% of patients admitted to intensive care units, a tracheostomy is performed [7]. While it is usually carried out between days 10 and 14 of intubation, the optimum timing of a tracheostomy has yet to be determined by evidence-based practice [8]. Previously conducted reviews found no advantage of early tracheostomy in terms of survival [9-12].

Complications from tracheostomy placement are uncommon, but they may be fatal. Patients that need a tracheostomy may have several comorbidities that increase the risk of severe complications such as hemorrhage, tracheal perforation, airway complications and may lead to mortality [13]. Per year, an estimated 500 people in the United States die or become permanently disabled as a result of tracheostomy [14]. Patients undergoing tracheostomy had a lower mortality rate than non-tracheostomy patients in a survey conducted by Freeman et al., which involved 43,916 chronically ill patients [15]. In our analysis where a larger number of tracheostomies were included from different studies, we have found a reduced in-hospital mortality rate in early tracheostomy patients by 2.6% which is significant. Patients who had late tracheostomy are less likely to be discharged home and more likely to be admitted to skilled nursing facility or intermediate care facility and more likely to end up on home health care. Despite the statistical significance of the outcome for disposition against medical advice, the proportions are so small that the results may be unreliable.

Prolonged length of hospital stay was linked to tracheostomy and postoperative infection, which may further increase the chance of hospital-related complications and outcomes [16]. In another observational review involving 10,927 patients, Scales et al. measured early (10 days) and late (10 days) tracheotomies and observed substantial decreases in unadjusted 90 days, one year, and study mortality in the early tracheotomy cohorts [17]. In a study where tracheostomy timing was seen as a consecutive parameter, Freeman et al. found that tracheotomy timing was associated significantly with mechanical ventilation time, ICU stays, and hospital stay [15]. We found to reduce hospital stay to an average of 27.7 days in early tracheostomy patients compared to late tracheostomy patients which involve 32.3 days on average. The average amount of hospital cost associated with a tracheostomy is around $265,499 [18]. Reduced ICU and hospital stays would result in lower ICU costs and medical resources. We also analyzed reduced hospitalization costs for early vs late tracheostomy which include $101,141 and $116,047, respectively.

Limitations

Our study has its own limitations. Registration bias is expected as it is a retrospective study, also it prohibits us from drawing any conclusions about the relationship between early tracheostomies and the reduction in postoperative morbidity and mortality. Furthermore, we did not conduct a formal cost study to validate the financial advantage of early tracheostomy on a societal scale, although decreases in the duration of mechanical ventilation, length of ICU stays, and hospital length of stay is always linked with significant fund savings and resource optimization. Although our study contains a large number of subjects, the overall number of patients who had tracheostomy is lower when compared to other research, which may raise eyebrows about the research's efficacy. At this point, we should emphasize that one of the most important variables limiting the number of patients included (particularly in the early tracheostomy group) family consent on the procedures, which delays early tracheostomy and transitions it into the late tracheostomy.

## Conclusions

This study indicates that despite the potentially life-threatening complications of a tracheostomy, our data clearly shows that early tracheostomy is associated with reduced in-hospital mortality, reduce the need for skilled nursing facility or home medical care, as well as direct variables, length of stay, and potentially overall hospital cost in the ICU. The benefits of early tracheostomy on in-hospital mortality were significant, and doctors should consider them carefully when determining the optimal time for tracheotomy. These findings provide important clinical implications and could lead to changes in the practice regarding the timing of tracheostomy performed in critically ill patients who require mechanical ventilation.
